# Tau abnormalities and microglial remodeling in glaucoma: potential links to phagocytosis-related injury

**DOI:** 10.3389/fneur.2026.1940010

**Published:** 2026-09-07

**Authors:** Chengdong Li, Ruijia Song, Ling Yu

**Affiliations:** Department of Ophthalmology, The Affiliated Hospital of Southwest Medical University, Southwest Medical University, Luzhou, Sichuan, China

**Keywords:** aqueous humor biomarkers, glaucoma, glaucoma-associated microglia, neuroimmune remodeling, phagocytosis-related injury, retinal ganglion cells, tau dysregulation

## Abstract

**Background:**

Glaucoma is a progressive optic neuropathy characterized by retinal ganglion cell degeneration and optic nerve damage. Increasing evidence indicates that glaucomatous injury is not limited to the retina, but may also involve downstream visual structures and neuroimmune responses. Tau abnormalities and microglial remodeling have each been reported in glaucoma models and human glaucomatous tissues, yet they are usually discussed as separate pathological findings. Whether these processes are connected, and how they might contribute to synaptic, dendritic, and axonal injury, remains insufficiently defined.

**Review focus:**

This review examines current evidence for tau dysregulation and glaucoma-associated microglial remodeling in glaucoma. Experimental studies support retinal tau accumulation, altered phosphorylation, and redistribution from axonal to somatodendritic compartments after intraocular pressure elevation, whereas evidence for oligomeric tau remains limited to a single rat ocular-hypertension study. In parallel, glaucoma-associated microglia show loss of homeostatic signatures and acquisition of neurodegeneration-associated features, including APOE/Galectin-3-related remodeling, complement activation, inflammatory signaling, and phagocytosis-related changes. Evidence from tauopathy research provides several plausible links between tau-related neuronal stress and microglial responses, including NF-κB signaling, TLR4–NLRP3 inflammasome activation, complement-mediated recognition, and glial clearance of tau-altered synaptic or neuronal substrates. However, most of these links have not been directly tested in glaucoma. Aqueous humor biomarkers, including total tau, phosphorylated tau, neurofilament light chain, glial fibrillary acidic protein, APOE, and Galectin-3, may offer clinically accessible readouts of neuronal and glial injury, but they cannot establish temporal order or causality.

**Conclusion:**

Available evidence supports tau dysregulation and glaucoma-associated microglial remodeling as relevant but not yet causally linked components of glaucomatous neurodegeneration. A cautious working model is that tau-related neuronal stress may alter dendritic, synaptic, or axonal substrates, while remodeled microglia and complement pathways may influence recognition and clearance of vulnerable structures. Future studies should test these interactions directly by combining tau species-specific analysis, microglial state profiling, engulfment assays, and visual pathway outcomes within the same experimental framework.

## Background

Glaucoma is a progressive optic neuropathy involving retinal ganglion cell (RGC) degeneration, optic nerve damage, and irreversible visual field loss (1). However, glaucomatous injury is not confined to the retina and optic nerve but may extend throughout the visual pathway (1, 2). Longitudinal diffusion MRI studies in primary open-angle glaucoma have demonstrated progressive degeneration involving both pre-geniculate and post-geniculate visual structures (2), supporting a broader view of glaucoma as a neurodegenerative disorder in which neuronal vulnerability and glial responses contribute to pathology across interconnected regions of the visual system (1, 2).

A possible human genetic link comes from a cross-ancestry genome-wide association study that identified a primary open-angle glaucoma risk locus encompassing *MAPT*. The same locus was associated with ganglion cell–inner plexiform layer and retinal nerve fiber layer thickness after correction for multiple testing (3). However, locus-level association does not identify the causal gene and cannot, by itself, establish a role for *MAPT* or demonstrate retinal tau pathology.

Glaucoma studies have identified changes in tau abundance, phosphorylation, and localization, with tau manipulation influencing RGC injury in experimental models (4–6). In parallel, ocular hypertension is accompanied by loss of homeostatic microglial programs, APOE/LGALS3-associated remodeling, and complement-related synaptic vulnerability (7–11).

Whether tau dysregulation and glaucoma-associated microglial remodeling are mechanistically connected remains unresolved. Direct evidence that tau abnormalities initiate or shape microglial phagocytic programs in glaucoma is lacking. Tauopathy studies implicate Galectin-3, microglial NF-κB, and TLR4–NLRP3 signaling as candidate routes by which tau-related stress could influence microglial behavior (12–14), whereas glaucoma studies independently support APOE/Galectin-3 remodeling and complement-associated synaptic vulnerability (7, 9, 11). Their mechanistic convergence in glaucoma remains to be demonstrated.

**Figure 1:**
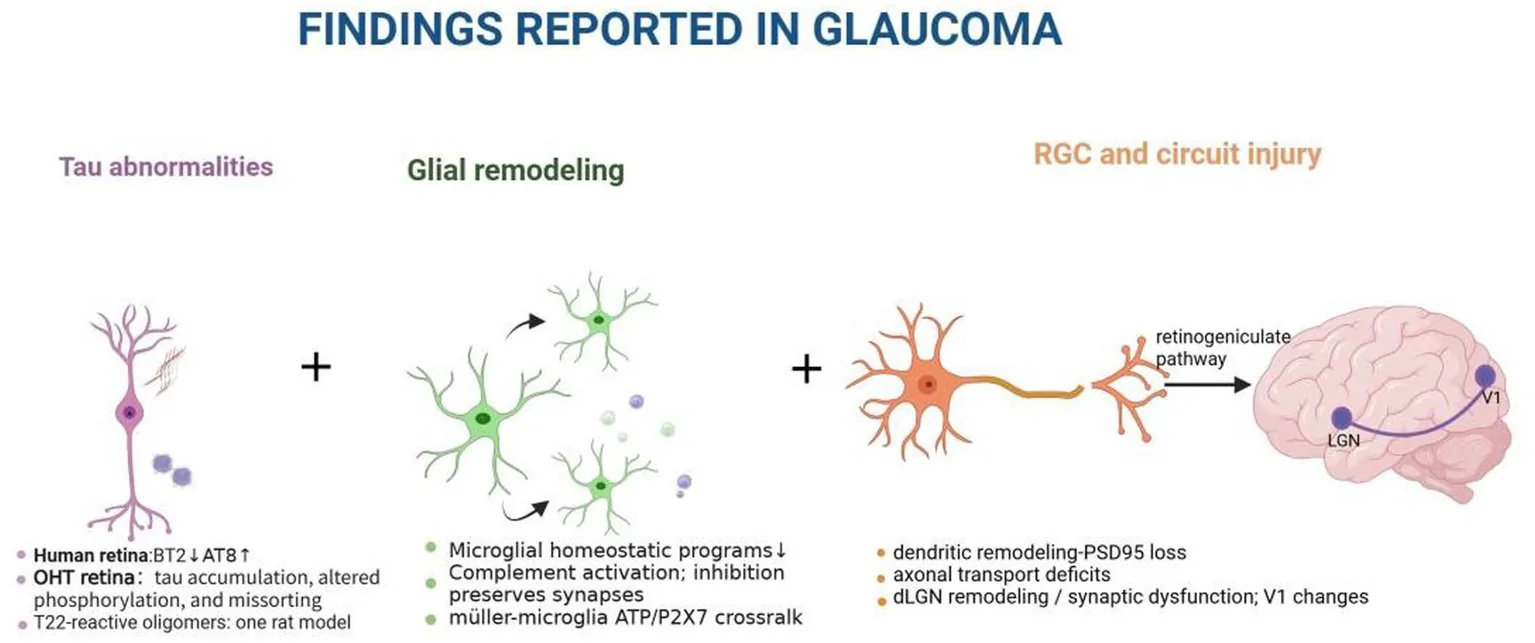
Findings reported in human glaucoma and experimental glaucoma models. Human glaucomatous retina shows reduced BT2 immunoreactivity and increased AT8 immunoreactivity, with AT8 staining localized predominantly to retinal horizontal cells; experimental ocular hypertension models show retinal tau accumulation, altered phosphorylation, somatodendritic missorting, and, in one rat model, T22-reactive oligomers. Glial findings include suppression of homeostatic programs in optic nerve head microglia, complement activation with preservation of early dendritic and synaptic architecture after classical pathway inhibition, and ATP/P2X7-dependent Müller cell–microglia crosstalk. Reported neuronal and circuit changes include RGC dendritic remodeling, loss of PSD95-positive synaptic input, axonal transport deficits, dLGN microglial remodeling and retinogeniculate synaptic dysfunction, and neuronal and visual-tuning changes in V1. The plus signs separate parallel evidence domains and do not imply a demonstrated causal sequence or tau–glia mechanistic link. The retinogeniculate arrow denotes anatomical connectivity and reported circuit dysfunction, not tau transfer or propagation.

This review examines the evidence supporting potential interactions among tau dysregulation, microglial remodeling, and phagocytosis-related injury in glaucoma. Figure 1 summarizes findings reported in human glaucoma and experimental glaucoma models across three parallel domains—tau abnormalities, glial remodeling, and RGC/circuit injury—without implying a demonstrated causal sequence or tau–glia mechanistic link. Aqueous humor biomarkers, including tau-related proteins and glial-response molecules, are discussed as clinically accessible indicators of ongoing neurodegenerative and neuroimmune processes rather than as evidence of causality (15, 16). Existing studies have usually focused separately on neuronal tau pathology and microglial inflammatory and phagocytic responses (4, 6–11). The mechanistic relationship between these processes remains poorly defined. This review therefore aims to synthesize current evidence, clarify the limits of existing causal inference, and identify priorities for future investigation.

### Literature search and evidence synthesis

A structured literature search was conducted in PubMed from database inception to May 2026. Separate searches combined terms related to glaucoma (“glaucoma,” “ocular hypertension,” and “optic nerve crush”) with terms covering tau biology (“tau,” “MAPT,” “phosphorylated tau,” “tau oligomer,” and “tauopathy”); glial and phagocytic responses (“microglia,” “astrocyte,” “Müller cell,” “APOE,” “galectin-3,” “complement,” “phagocytosis,” “phosphatidylserine,” and “NLRP3”); central visual pathways (“lateral geniculate nucleus,” “visual thalamus,” “retinogeniculate,” and “visual cortex”); and human evidence (“GWAS,” “aqueous humor,” “serum,” “plasma,” and “neurofilament light”). Additional searches paired tau-related terms with retinal or cerebral tauopathy, glial uptake, synapse elimination, and tau propagation. Searches were restricted to English-language publications, and the reference lists of relevant original studies and reviews were examined to identify additional articles.

Peer-reviewed human studies, experimental glaucoma or ocular hypertension studies, optic nerve crush studies providing mechanistic context, and retinal or cerebral tauopathy studies relevant to the proposed tau–glia framework were considered. Reviews were used to establish background and identify additional primary studies. Articles focused solely on intraocular pressure-lowering treatment, epidemiology, or biomarkers unrelated to tau biology, glial responses, or neurodegeneration were excluded. Because this article is a narrative perspective rather than a systematic review, no formal risk-of-bias assessment or quantitative evidence grading was performed. Table 1 therefore organizes evidence according to its source and relevance to glaucoma rather than assigning evidence strength or causal status.

**Table 1:** Evidence map and key boundaries of the proposed tau–microglia framework in glaucoma.

Evidence source	Key evidence	Supported inference	Main limitation	Reference(s)
Human genetics	*MAPT*-region POAG locus associated with GCIPL/RNFL thickness.	Human genetic link to the *MAPT* region.	Causal gene and retinal tau pathology remain unproven.	(3)
Glaucoma: tau	Human BT2 reduction and AT8 elevation; OHT-related tau accumulation, missorting, and T22-reactive oligomers; protection with tau silencing.	Tau dysregulation may contribute to glaucomatous RGC injury.	Human data are observational; oligomeric tau was detected in one OHT model, whereas seed competence remains untested.	(4–6)
Glaucoma: microglia	Loss of homeostatic programs; *Apoe*/*Lgals3* remodeling; neuroprotection after pathway modulation.	Microglial states modify glaucoma progression.	Tau dependence and a uniform microglial state remain unproven.	(7, 8, 10)
Glaucoma: macroglia	Müller-cell ATP/P2X7R crosstalk; early phagocytic astrocyte responses.	Macroglia contribute to inflammation and remodeling.	Links to tau and selective RGC engulfment are unknown.	(17, 18)
Complement	C1q upregulation; protection by complement inhibition; ANX007 target engagement.	Complement is modifiable and clinically targetable.	Human efficacy and tau→complement ordering remain unproven.	(9, 11, 19, 20)
PS-mediated clearance	Acute OHT: PLSCR1-dependent PS exposure and clearance of apoptotic RGCs.	Pressure injury can trigger PS-mediated RGC clearance.	Does not show tau-dependent phagoptosis of viable RGCs.	(21)
Central visual pathway	LGN tau/Aβ pathology, retinogeniculate dysfunction, dLGN microglial remodeling, and V1 changes.	Glaucoma affects central visual pathways.	Tau transfer/seeding is unproven; amyloid co-pathology complicates inference.	(22–25)
Human biomarkers	Aqueous total tau, NfL, APOE/Galectin-3, and complement alterations.	Candidate neuronal and glial biomarkers.	Origin, specificity, timing, and causality remain unresolved.	(15, 26–28)
ONC (contextual)	Tau silencing or CDK5/GSK3β inhibition reduces phosphorylated tau and/or RGC loss.	Tau pathways influence acute axonal injury.	Acute injury kinetics limit extrapolation to chronic glaucoma.	(29–31)
Retinal tauopathy (contextual)	RGC pS396 and T22-reactive tau associated with RGC loss, retinal microglial p-tau, and SC and V1 p-tau after subretinal AD lysate injection.	Retinal tau–glia interactions are biologically plausible.	AD and transgenic models differ from glaucoma, and seed transport was not directly traced	(32–34)
Cerebral tauopathy (extrapolated)	Galectin-3 signaling, neuronal PS exposure, and glial engulfment of tau-bearing neurons and synapses	Plausible tau-to-glia recognition pathways.	Not demonstrated in glaucomatous RGCs.	(12, 13, 35, 36)

This table is a narrative evidence map rather than a formal evidence-grading system. Evidence from human glaucoma and experimental OHT/glaucoma models is distinguished from contextual evidence derived from ONC and non-glaucomatous retinal tauopathy studies and from mechanisms extrapolated from cerebral tauopathy. These categories indicate the source and relevance of the evidence to glaucoma, rather than its strength or causal status. Citations are representative rather than exhaustive. Differences in age, sex, disease stage, species or strain, experimental model, tissue, and assay should be considered when comparing studies.

## Tau–microglia evidence and proposed framework

### Main evidence for tau abnormalities in glaucoma

Tau abnormalities in glaucoma are manifestations of neuronal stress and dysfunction rather than evidence that glaucoma represents a retinal form of classical tauopathy. Under physiological conditions, tau maintains microtubule stability, preserves axonal architecture, and supports neuronal compartmentalization; disruption of these functions make long-projecting neurons vulnerable to injury (37, 38). This biological concept is particularly relevant to RGCs, the degeneration of which in glaucoma is associated with axonal stress, cytoskeletal instability, and impaired neuronal maintenance. Current glaucoma-related evidence is strongest for altered tau abundance, phosphorylation, and somatodendritic missorting. Oligomeric tau is less extensively characterized, and the evidence remains stronger for neuronal involvement than for tau-driven glial remodeling (4–6).

Proteomic analyses place these tau alterations within a broader landscape of neurodegenerative and stress-related changes in glaucomatous retina and vitreous, without resolving the specific tau species involved (5, 39).

Current glaucoma studies do not resolve all tau species to the same extent. In the cited human glaucoma study, reduced BT2 immunoreactivity and increased AT8 staining indicated altered tau abundance and phosphorylation but not the presence of oligomeric tau; AT8 immunoreactivity was observed predominantly in retinal horizontal cells rather than RGCs (5). This localization should be regarded as a study-specific finding rather than a general feature of human retinal tau pathology. By contrast, T22-reactive tau oligomers were detected alongside altered phosphorylation and somatodendritic missorting in a rat model of ocular hypertension (4). This provides direct, but model-specific, evidence of tau oligomerization and does not establish that comparable assemblies occur in human glaucomatous RGCs or possess seeding activity. AAV9-mediated tau modulation further showed that altering tau abundance affects retinal injury and survival signaling, although measurements of total tau and phosphorylation at Ser199/202 and Ser404 did not identify the species responsible for these effects (6). Thus, oligomeric tau has limited experimental support in glaucoma, whereas extracellular and seeding-competent tau remain unproven, leaving their proposed roles in microglial recognition unresolved.

In human MCI/AD retina, pS396-tau and T22-reactive tau oligomers have been detected in RGCs and are associated with RGC loss, while PHF-1- and AT8-positive aggregates have been localized within retinal microglia, consistent with p-tau uptake (32, 33). In TAU58 mice, subretinal injection of AD brain lysates increased retinal p-tau pathology and was accompanied by greater p-tau immunoreactivity in the contralateral superior colliculus and the internal pyramidal layer of V1. Because the injected tau was not directly traced, these changes are consistent with, but do not establish, retina-to-brain propagation (34). More broadly, human retinal tau localization is heterogeneous across disease contexts, retinal layers, tau species, and pathological stages; a large postmortem series described a four-stage pattern of p-tau distribution involving the outer plexiform, inner nuclear, and inner plexiform layers (40).

Experimental ocular hypertension models also provide mechanistic support for tau’s role in pressure-related retinal injury. In a rat ocular-hypertension model, elevated intraocular pressure was accompanied by retinal tau accumulation, altered phosphorylation, somatodendritic missorting, and T22-reactive oligomeric tau (4). These changes are biologically significant because RGCs rely heavily on cytoskeletal stability and efficient axonal transport. Retinal tau and mutant tau models have demonstrated that tau accumulation, hyperphosphorylation, and aggregation can impair RGC function and axonal transport before substantial neuronal loss becomes apparent (41, 42). Within this context, tau abnormalities are best viewed not as passive byproducts of neurodegeneration but as molecular disturbances that may lower the threshold for RGC dysfunction under chronic glaucomatous stress. The strongest evidence for the functional relevance of tau comes from studies that directly manipulate its expression. Tau-targeted siRNA reduces both somatic and axonal RGC injury in experimental glaucoma, suggesting that tau reflects not only ongoing damage but also disease severity (4). Findings from AAV9-mediated tau modulation further strengthen this interpretation. Tau silencing attenuates inner retinal degeneration and enhances Akt/Erk survival signaling, whereas tau overexpression exacerbates retinal injury under glaucomatous conditions (6). Additional support is provided by acute optic nerve injury models, in which tau silencing or inhibition of GSK3β and Cdk5 reduces phosphorylated tau formation and improves RGC survival following optic nerve crush (29–31). Although ONC differs from chronic glaucoma in injury kinetics and mechanism, these studies provide complementary mechanistic evidence that kinase-dependent tau phosphorylation contributes to RGC vulnerability after axonal stress.

Tau-related abnormalities may extend beyond the retina, but this evidence requires particular caution. In this review, tau transfer denotes movement between cells or anatomical regions, whereas spread refers more broadly to dissemination across connected sites. Propagation is reserved for evidence that transferred, seeding-competent tau induces templated pathology in recipient cells. The detection of phosphorylated tau in a connected region is therefore insufficient, by itself, to demonstrate propagation. In a rhesus monkey model of chronic ocular hypertension, amyloid-β deposition and phosphorylated tau-related signals have been reported within the lateral geniculate nucleus (LGN) (23). Such findings are consistent with growing recognition that glaucoma can affect multiple levels of the visual pathway and are supported by proteomic evidence implicating neurodegeneration-associated pathways in glaucomatous ocular tissues (23, 39). Nevertheless, these observations should not be interpreted as evidence of trans-synaptic tau propagation from the retina to the brain. A more cautious interpretation is that chronic ocular hypertension may be associated with tau-related molecular abnormalities at both retinal and central visual system levels without establishing a specific tau-spreading mechanism.

Overall, glaucoma studies support altered and experimentally modifiable tau biology, whereas extracellular or seeding-competent tau and tau-driven microglial remodeling remain unproven (4–6, 39).

### APOE/LGALS3-associated microglial remodeling and phagocytosis-related priming in glaucoma

Microglial alterations in glaucoma are more appropriately viewed as changes in cellular state than as nonspecific activation. Under physiological conditions, microglia continuously survey the neural environment, support neuronal homeostasis, and maintain local synaptic environments, whereas microglia under neurodegenerative stress can undergo substantial transcriptional reprogramming, adopting molecular profiles enriched in lipid metabolism, immune signaling, complement activity, and phagocytosis-related pathways (43, 44). In glaucoma, the critical issue is not simply whether microglia become reactive but whether they shift from their normal homeostatic surveillance toward remodeled states that alter their interactions with RGCs and their axons. Human glaucomatous optic nerve heads contain HLA-DR-positive microglia, while chronic glaucoma models demonstrate early microglial redistribution within the retina and optic nerve head before substantial structural degeneration becomes apparent (45, 46). Furthermore, ocular hypertension suppresses homeostatic and sensome-related gene expression in optic nerve head microglia, suggesting that the early microglial response in glaucoma includes not only increased inflammatory activity but also impaired environmental sensing (10).

Although glaucoma-associated microglia share selected features with disease-associated microglia (DAM) described in Alzheimer disease and other neurodegenerative disorders, these states are not equivalent. The reported overlap includes loss of homeostatic signatures together with increased expression of APOE-, Galectin-3-, and complement-associated programs (7, 43, 44). In both experimental glaucoma and human glaucomatous retina, elevated Apoe and Lgals3 expression places microglia within a neurodegenerative disease-like state, while modulation of the APOE/Galectin-3 axis influences the extent of neuronal loss (7). Moreover, restoration of pro-homeostatic microglial programs, including through IGFBPL1-mediated signaling, reduces neuroinflammation and attenuates glaucomatous neurodegeneration (8). Thus, glaucoma involves not only microglial “activation” but also a shift in microglial identity, characterized by weakened homeostatic sensing and increasing dominance of neurodegeneration-associated immune and metabolic programs.

In this review, phagocytosis refers broadly to the engulfment of neuronal material, regardless of whether the substrate is viable, dying, or already fragmented. Phagoptosis is used more narrowly for engulfment that causes the death of a cell that was viable when recognized. Accordingly, phagocytosis-related markers or complement activation do not, by themselves, establish phagoptosis.

Complement activation provides a bridge between microglial state remodeling and the possibility of phagocytosis-related injury. In glaucoma, retinal C1q is upregulated, C3aR1 promotes optic nerve degeneration, and inhibition of the classical complement pathway preserves early dendritic and synaptic architecture (9, 11, 19). These observations position complement as a marker of inflammation and potential mechanism through which glial cells can target stressed neuronal structures. Nevertheless, complement activation should not automatically be equated with pathogenic phagocytosis. Complement proteins are involved in diverse biological processes, including debris clearance, synaptic remodeling, and tissue repair, and their functional significance depends heavily on disease stage and the nature of the targeted structures (9, 11, 19). Consequently, complement alterations in glaucoma indicate a phagocytosis-permissive microenvironment rather than direct proof that microglia are actively eliminating viable RGCs or their processes.

C1q inhibition has also entered clinical testing in glaucoma. In phase Ia/Ib studies involving 26 patients with primary open-angle glaucoma, intravitreal ANX007 was well tolerated and achieved complete ocular C1q target engagement 4 weeks after dosing (20). The phase I program addressed safety and pharmacodynamic engagement; it was not designed to demonstrate neuroprotective efficacy or determine whether complement activation lies downstream of tau. This distinction becomes particularly important when interpreting commonly used phagocytosis-related markers. Molecules such as CD68, CLEC7A, Galectin-3, and complement-associated proteins may reflect enhanced lysosomal activity, immune remodeling, neurodegeneration-associated state transitions, or increased phagocytic capacity but do not independently demonstrate pathological engulfment of intact synapses, axons, or neuronal cell bodies (7, 9, 11, 19, 43, 44). The role of microglia in glaucoma is therefore unlikely to fit a simple protective-versus-harmful paradigm. Partial suppression of microglial activity with minocycline improves optic nerve integrity in DBA/2 J mice, whereas broad or prolonged microglial depletion can increase susceptibility to ocular hypertension-induced injury and exacerbate RGC loss (47–49). A more mechanistic interpretation is that the impact of microglia depends on cellular state, timing, local environmental conditions, and the specific neuronal substrates involved.

Together, these findings support microglial state remodeling and a phagocytosis-permissive environment in glaucoma, but not a tau-initiated phagocytic cascade.

### Müller glia, astrocytes, and glial crosstalk in glaucoma

Microglial remodeling occurs within a multicellular glial environment. In a mouse model of chronic ocular hypertension, activated Müller glia released ATP and promoted P2X7 receptor-dependent microglial activation. The resulting increase in microglial TNF-α and IL-6 further enhanced inflammatory gene expression in Müller glia, establishing a reciprocal inflammatory loop (17). These findings provide direct evidence that Müller glia can shape microglial responses in experimental glaucoma. However, this study did not examine tau, complement-dependent target recognition, or the engulfment of RGC structures. Müller glia should therefore be considered potential regulators of the inflammatory environment in which injured or tau-altered RGC compartments are encountered, rather than established mediators of tau-dependent phagocytosis.

Astrocytes occupy a different anatomical interface. At the optic nerve head, their processes directly contact the unmyelinated and metabolically demanding segments of RGC axons and respond early to IOP-related stress (18, 50). In a microbead-induced ocular hypertension model, optic nerve head astrocytes showed heterogeneous upregulation of Gulp1 and Lgals3 and increased incorporation of axonal mitochondria, consistent with enhanced transmitophagy in a subset of astrocytes (18). This work demonstrates an early astrocytic phagocytic response, but the measured substrate was axonal mitochondria. It does not establish engulfment of intact axons, synapses, or viable tau-bearing RGCs, nor does it determine whether the response is protective or detrimental. Complement inhibition preserves RGC dendrites and synapses in experimental glaucoma, but the responsible effector cell has not been assigned specifically to astrocytes or Müller glia (11). Müller glia and astrocytes may therefore influence inflammatory amplification, axonal maintenance, and the local conditions governing cellular clearance. Whether either cell type recognizes tau-altered substrates or participates in tau-dependent synaptic elimination in glaucoma remains unresolved.

### Applicability and limits of AD/tauopathy-derived mechanisms in glaucoma

Evidence from AD is derived from an amyloid-rich brain environment (36, 51). By contrast, the P301S model cited here makes mutant tau expression a primary experimental feature (35). In contrast, glaucomatous injury arises in a distinct anatomical and biomechanical context. Mechanical strain related to intraocular pressure is focused at the optic nerve head, where retinal ganglion cell axons pass through an unmyelinated region with high metabolic demand before continuing into the myelinated optic nerve (1, 50). Although amyloid beta and phosphorylated tau signals have been reported in the lateral geniculate nucleus of an ocular hypertensive monkey model, this finding does not establish amyloid-related co-pathology as a general feature of human glaucoma (23). Tau-related changes in glaucoma may therefore arise downstream of stress related to pressure and axonal injury and modify neuronal vulnerability without constituting the initiating lesion.

Findings from tauopathy research remain valuable for identifying candidate cellular pathways, but the specific tau species and neuronal substrates involved cannot be assumed to be shared across diseases. Glaucoma studies support altered tau abundance and phosphorylation in human specimens, together with tau accumulation, somatodendritic missorting, and functional effects of tau manipulation in experimental models (4–6). T22-reactive oligomers have been detected in only one ocular hypertension model (4), whereas the existence of extracellular, seeding-competent tau in glaucoma remains unproven.

Glaucoma has also been linked to microglial remodeling involving APOE and Galectin-3, as well as to synaptic vulnerability driven by complement (7, 9, 11, 19). Whether either process lies downstream of tau remains untested. The distinction is particularly clear for phosphatidylserine exposure. In a P301S tau model, viable neurons containing tau filaments were shown to expose phosphatidylserine prior to microglial engulfment (35). By contrast, an acute ocular hypertension study linked PLSCR1-dependent phosphatidylserine exposure to RGC apoptosis and subsequent microglial clearance (21). No glaucoma study has demonstrated phosphatidylserine exposure on viable tau-positive RGCs or their selective engulfment. Likewise, synaptic elements containing tau oligomers were found within microglia and astrocytes in postmortem AD visual cortex, but not in glaucomatous retina (36).

Accordingly, the following section treats mechanisms derived from AD and tauopathy research as testable hypotheses rather than established glaucoma pathways.

### A testable tau–microglia hypothesis in glaucoma

Any attempt to link tau abnormalities with microglial phagocytosis in glaucoma is a staged and multifactorial process rather than a single linear pathway. Current evidence from glaucoma research supports three parallel observations: ocular hypertension alters retinal tau abundance, phosphorylation, and somatodendritic localization; glaucoma-associated microglia undergo APOE/Galectin-3-associated disease-like remodeling; and complement-dependent mechanisms contribute to early dendritic and synaptic vulnerability (4, 7, 9, 11, 19). Although these findings are biologically compatible, their temporal relationships and causal interdependencies remain unresolved. A useful working framework therefore posits that tau-mediated neuronal stress alters the properties of RGCs and their associated synaptic structures, glaucoma-associated microglial remodeling generates a permissive neuroimmune environment, complement and related recognition pathways determine target selection, and the resulting phagocytic response ranges from beneficial clearance to pathological elimination of neural structures (4, 7, 9, 11, 19, 51).

At the neuronal level, tau abnormalities may alter the substrates that microglia survey. Tau missorting from axonal compartments to somatodendritic regions is particularly relevant in glaucoma because RGCs depend on highly organized cytoskeletal architecture and efficient long-range axonal transport (4). Whether such tau-related changes generate glial-recognition signals in glaucoma remains unknown. These observations provide a biologically plausible framework in which phosphorylated, oligomeric, or mislocalized tau converts neuronal stress into structural and molecular alterations affecting dendritic, synaptic, and axonal surfaces, thereby increasing the likelihood of microglial recognition. Within this context, tau functions less as a direct extracellular inflammatory mediator and more as a determinant of neuronal substrate integrity.

Whether such stressed neuronal substrates are preserved, repaired, or eliminated is likely to depend on the surrounding microglial state. In this regard, APOE/Galectin-3-associated microglial remodeling assumes particular importance. Experimental and human studies consistently demonstrate neurodegeneration-associated phenotypic changes in retinal microglia, and manipulation of the APOE/Galectin-3 axis directly influences neuronal survival (7). Findings from tauopathy research provide additional mechanistic context. Galectin-3 promotes microglial activation and tau propagation, NF-κB signaling enhances tau-associated toxicity and spread, and pathological tau can activate MyD88- and NLRP3–ASC-dependent inflammatory pathways in myeloid cells (13, 14, 52). Rather than representing competing explanations, these pathways may constitute complementary components of a permissive neuroimmune environment. APOE/Galectin-3-associated remodeling may weaken homeostatic regulation, NF-κB signaling may amplify inflammatory tone, and inflammasome activation may translate tau-induced stress into downstream effector responses. Importantly, the strength of evidence differs substantially among these mechanisms. APOE/Galectin-3-associated remodeling is directly supported by glaucoma studies, whereas the roles of NF-κB and NLRP3 signaling remain largely inferred from tauopathy literature (7, 13, 14, 52).

Target selection represents the next critical stage of the proposed model. Activated microglia do not indiscriminately engulf neural structures. Effective phagocytosis requires the integration of target-derived signals, opsonization, and receptor-mediated recognition. Phosphatidylserine exposure, complement deposition, and phagocytic receptors may contribute through distinct mechanisms (51). Among these pathways, complement currently provides the most compelling mechanistic bridge between neuronal stress and microglial recognition in glaucoma. Retinal C1q upregulation, the contribution of C3aR1 signaling to optic nerve degeneration, and the protective effects of classical complement inhibition on dendritic and synaptic integrity support this interpretation (9, 11, 19).

Evidence from tauopathy research further expands this framework. In human AD tissue, tau oligomer-containing synaptic elements were preferentially engulfed by microglia and astrocytes (36). Work in P301S-tau neurons identified a different route: viable neurons bearing tau filaments exposed phosphatidylserine before microglial engulfment (35). Extracellular tau may also act on microglia, stimulating the phagocytosis of viable neurons through TLR4–NLRP3–caspase-1 signaling (12). Taken together, these findings point to a scenario in which tau-related alterations in neuronal and synaptic surfaces can lower the threshold for glial recognition, potentially interacting with complement tagging and other “eat-me” signals in shaping which RGC-associated structures become vulnerable to engulfment. Even so, whether similar tau-driven processes occur in glaucoma, particularly at the level of RGC dendrites, synapses, or axons, has yet to be demonstrated.

The consequences of phagocytosis are similarly dependent on biological context. Clearance of cellular debris and irreversibly damaged synaptic material may limit inflammation and preserve tissue integrity, whereas excessive engulfment of stressed but potentially recoverable neural structures could amplify neurodegeneration (12, 35, 51). This distinction is particularly relevant in glaucoma, where early pathological changes frequently manifest as dendritic and synaptic dysfunction before substantial loss of RGC somata becomes evident. A phagocytosis-permissive microglial state may therefore contribute to disease progression by progressively eroding neuronal connectivity and reducing axonal output rather than by directly inducing widespread neuronal death. At the same time, indiscriminate suppression of phagocytic activity may be equally detrimental if it impairs debris clearance and disrupts tissue homeostasis. Consequently, the central question is not whether phagocytosis occurs, but rather which structures are removed, when removal occurs, and under what microenvironmental conditions.

Microglial interactions with tau introduce the possibility of a feedback mechanism, although this aspect of the model remains highly speculative in glaucoma. Experimental studies have demonstrated that microglia can internalize tau oligomers and fibrils, with the biological consequences of uptake depending on tau species, receptor engagement, intracellular degradation capacity, and exosome-mediated release (53–55). Under certain circumstances, tau uptake appears beneficial; for example, complement receptor 4 has been implicated in the microglial clearance of extracellular tau fibrils (55). Under other conditions, microglia may facilitate tau propagation, as both microglial depletion and inhibition of exosome production reduce tau spread in experimental tauopathy models (53). To date, however, no glaucoma study has demonstrated microglial internalization of retinal tau, transport of tau seeds along the visual pathway, or the existence of a sustained tau–microglia feed-forward cycle. Accordingly, this proposed feedback mechanism should be regarded as a hypothesis informed by tauopathy biology rather than as an established component of glaucomatous neurodegeneration.

This framework leads to several experimentally testable predictions. An upstream role for tau would predict that confirmed tau modulation alters at least one downstream response, including *Apoe/Lgals3* induction, complement deposition, or preferential engulfment of tau-altered RGC compartments (4, 6, 7, 9, 11, 19). Persistence of these responses after adequate tau manipulation would instead favor a parallel model; tau might still influence neuronal vulnerability, but its role would be more restricted. Complement inhibition offers a particularly informative test. Preservation of dendrites or synapses without a corresponding benefit from tau reduction would be more consistent with complement acting independently of tau, possibly as a convergent effector of pressure- and axon-related injury (6, 9, 11, 19). Phagoptosis requires a more specific form of evidence: phosphatidylserine exposure on viable, tau-altered RGC structures before engulfment. Engulfment confined to apoptotic elements, or the absence of preferential recognition of tau-bearing substrates, would not support tau-dependent phagoptosis (12, 21, 35).

### Synaptic vulnerability and circuit remodeling in the proposed tau–microglia framework

Experimental glaucoma models demonstrate that dendritic remodeling, loss of PSD95-positive synaptic input, and deficits in axonal transport can occur before or alongside overt RGC loss, although their timing varies among models and RGC types (56–62). Classical complement inhibition preserves early dendritic and synaptic architecture, indicating that these compartments are modifiable components of glaucomatous injury (11). Dendrites, synapses, and axons therefore provide relevant anatomical substrates for evaluating the proposed interaction between neuronal stress and glial responses.

Experimental tests of a tau–microglia interaction should extend beyond RGC survival. Dendritic architecture, synaptic integrity, axonal transport, and retinal function should be assessed alongside tau species and microglial state within the same model, allowing compartment-level injury to be related temporally to later neuronal loss (56–62). Substrate-specific engulfment assays would further distinguish structural vulnerability from actual glial removal of tau-altered material.

Consideration of downstream visual structures, including the LGN and primary visual cortex (V1), should therefore be framed within the context of impaired retinal output rather than as a separate extension of the hypothesis. The dorsal LGN (dLGN) occupies a particularly important position because it receives direct input from RGC axons and is therefore likely to reflect disturbances in axonal transport and retinogeniculate transmission (24, 60). Evidence of microglial remodeling within the visual thalamus further suggests that altered retinal input can elicit both neuronal and glial responses in downstream relay nuclei (22). Accordingly, the dLGN may represent the earliest circuit-level site at which retinal connectivity failure becomes anatomically and biologically evident rather than an independent pathological process disconnected from retinal disease.

Interpretation of V1 involvement requires greater caution. Experimental studies have reported neuronal loss and altered visual tuning in V1 of glaucoma models (25). However, these observations do not justify claims of direct tau propagation or microglia-mediated disease transmission along the retino-geniculo-cortical pathway. A more conservative interpretation is that tau-related and complement-associated retinal injury compromises dendritic and synaptic integrity, resulting in impaired RGC output and disrupted retinogeniculate signaling. Over time, persistent alterations in visual input may induce secondary remodeling within both the LGN and V1 (11, 22, 24, 25, 60). Anterograde trans-synaptic degeneration offers a plausible mechanism through which impaired retinal output could contribute to these downstream changes (63). Consistent with this interpretation, a recent UK Biobank MRI study found associations between RNFL and GCL thinning and structural abnormalities in primary visual regions (64). However, because the available human evidence is largely cross-sectional, it cannot establish that all cerebral abnormalities in glaucoma arise exclusively through this mechanism. Accordingly, anterograde trans-synaptic degeneration can be considered a potential downstream consequence of impaired RGC output within the broader tau–microglia framework, rather than evidence of retina-to-brain tau propagation.

### Aqueous humor biomarkers as clinical readouts

Aqueous humor should be regarded primarily as a clinically accessible source of information regarding intraocular neurodegenerative and neuroimmune processes rather than as evidence that a causal tau–microglia pathway has been established in patients. Tau-related proteins, including total tau (t-Tau) and phosphorylated tau (p-tau181), are detectable in ocular fluids (16, 65), and multiple studies have reported associations between aqueous humor neurodegeneration markers and structural or functional measures such as retinal nerve fiber layer (RNFL) thickness and visual field performance (15, 28, 66, 67). These findings suggest that aqueous humor may reflect ongoing neuronal stress, axonal injury, and glial responses within the eye. However, such measurements cannot determine whether tau abnormalities precede microglial remodeling, identify the cellular origin of detected proteins, or establish whether these molecules directly participate in phagocytosis-related injury.

The value of aqueous humor becomes more apparent when tau-related proteins are evaluated in conjunction with markers of glial and neuroimmune activity. APOE and Galectin-3, both strongly associated with disease-related microglial programs in glaucoma models, are detectable in aqueous humor and have been reported to be elevated in glaucoma (27, 68). Likewise, proteomic and cytokine studies have identified complement-related and inflammatory alterations that parallel mechanisms discussed elsewhere in this review (26, 69, 70). Nevertheless, caution remains necessary. These biomarkers should not be interpreted as direct surrogates of posterior-segment microglial phenotypes. Instead, they are more appropriately viewed as indicators of a broader intraocular molecular environment in which neuronal injury, glial responses, and inflammatory remodeling occur concurrently.

Interpretation of existing aqueous humor studies is further complicated by important methodological limitations. Most available datasets derive from cross-sectional surgical cohorts, frequently using cataract patients as control subjects, and are influenced by factors including sample volume, glaucoma subtype, disease severity, prior treatment exposure, and assay sensitivity (16, 71). Quantification of low-abundance phosphorylated tau species is particularly susceptible to platform-dependent variability. Consequently, single-marker approaches are unlikely to capture the complexity of a potential tau–microglia interaction. Future translational studies would benefit from integrated biomarker panels incorporating tau-related proteins, neurofilament light chain (NfL), glial fibrillary acidic protein (GFAP), APOE, Galectin-3, and selected complement- and inflammation-associated molecules. Correlating these molecular profiles with OCT, OCTA, visual field assessments, and, where feasible, tissue-based validation could provide a more comprehensive understanding of disease biology (15, 16, 26–28, 65–71). When applied in this manner, aqueous humor biomarkers may help determine whether neuronal injury and neuroimmune remodeling coexist within the same clinical setting while appropriately reserving questions of causality for experimental investigation.

### Knowledge gaps and future directions

A major limitation of the current tau–microglia framework is not the absence of individual markers but rather the lack of sufficient temporal and spatial resolution. Experimental glaucoma studies have shown altered retinal tau abundance, phosphorylation, and localization, whereas evidence for oligomeric tau remains model-specific. Separately, optic nerve head microglia can lose homeostatic and sensome-associated signatures before overt neurodegeneration (4, 10). The next step is to determine whether tau pathology and microglial remodeling occur sequentially, concurrently, or develop within distinct neuroanatomical compartments. Addressing these questions will require longitudinal investigations spanning the retina, optic nerve head, distal optic nerve, LGN and V1, with tau species and microglial phenotypes examined simultaneously within the same experimental paradigm. Similarly, APOE/Galectin-3-associated retinal microglial remodeling and optic nerve microglial responses should be investigated in conjunction with tau alterations rather than as separate pathological processes (7, 72). Emerging single-cell and spatial multi-omics technologies offer powerful tools for characterizing disease-relevant cellular states; however, their greatest value will lie in addressing a clearly defined mechanistic question: when and where tau-associated neuronal stress becomes linked to microglial remodeling (73).

A second unresolved issue is whether glaucoma-associated microglia actually engulf tau-altered neuronal substrates. Complement inhibition preserves early dendritic and synaptic structures in glaucoma, making dendrites and synapses plausible sites where neuronal stress is converted into glial recognition (11). However, complement deposition, increased CD68 expression, CLEC7A upregulation, and elevated Galectin-3 expression do not, by themselves, demonstrate the elimination of viable RGC structures (7, 11, 51). The key experiment is substrate-specific: do glaucoma-associated microglia preferentially engulf dendritic, synaptic, or axonal compartments that contain abnormal phosphorylated tau, oligomeric tau, or mislocalized tau? Tauopathy studies show that tau oligomer-containing synapses can be preferentially engulfed by glial cells, and tau-bearing neurons may expose phosphatidylserine signals that facilitate phagocytic recognition (35, 36). These mechanisms provide a useful hypothesis for glaucoma, but they must be tested directly in ocular hypertension models using engulfment assays, complement or phagocytic receptor manipulation, and tau modulation within the same design. Age and sex are additional sources of variability in the available evidence. Aging alters retinal microglial morphology and the expression of CD68, MHC-II, and P2RY12 in experimental ocular hypertension, while retinal tau pathology in non-glaucomatous tauopathy models also evolves with age (42, 74). Sex may likewise influence the initiating injury: male and female rats differ in IOP exposure, structural loss, and retinal function in chronic glaucoma models (75). These differences could affect tau burden or glial remodeling indirectly by altering the baseline tissue state and severity of injury. Evidence from cerebral tauopathy further indicates that sex cannot be assumed to be biologically neutral. Microglial miRNA and REV-ERBα pathways have shown sex-dependent effects on tau accumulation and inflammatory responses, although a longitudinal P301S study found comparable AT8, IBA1, and CD68 changes in males and females (76–78). Human AD neuropathology also suggests that the relationship between microglial activation and tau burden may differ by sex, but the amyloid-rich disease context limits direct extrapolation to glaucoma (79). Several key glaucoma studies used a single sex or a restricted age range, and the available datasets were generally not designed to test age-by-disease or sex-by-disease interactions. Age and sex should therefore be regarded as potential modifiers of IOP exposure, tau burden, glial state, and injury severity; whether either specifically alters the tau–glia relationship in glaucoma remains unresolved. Future studies should include both sexes and relevant age groups, report IOP exposure separately, and prespecify interaction analyses where feasible.

## Conclusion

Glaucoma studies document altered retinal tau abundance, phosphorylation, and somatodendritic distribution, with functional effects following tau manipulation in experimental models, whereas evidence for oligomeric tau remains limited and model-specific (4–6). Glaucoma-associated microglia show a loss of homeostatic programs and remodeling associated with APOE and LGALS3 (7, 8, 10), while complement studies support early dendritic and synaptic vulnerability as a modifiable component of injury (9, 11, 19). What remains unproven is a causal sequence in which tau abnormalities drive microglial target recognition and pathological engulfment of viable RGC compartments; the relevant recognition and engulfment mechanisms are supported mainly by tauopathy models rather than glaucoma (12, 35, 36). Resolving this question will require experiments that manipulate tau and microglial/complement pathways within the same ocular hypertension model while tracking tau species, substrate-specific engulfment, dendritic and synaptic integrity, axonal function, and RGC survival over time.

## Glossary

AAV9: Adeno-associated virus serotype 9
APOE: Apolipoprotein E
ASC: Apoptosis-associated speck-like protein containing a CARD
C1q: Complement component 1q
C3aR1: Complement C3a receptor 1
CD68: Cluster of differentiation 68
CLEC7A: C-type lectin domain containing 7A
Cdk5: Cyclin-dependent kinase 5
CR3: Complement receptor 3
CR4: Complement receptor 4
DAM: Disease-associated microglia
dLGN: Dorsal lateral geniculate nucleus
GFAP: Glial fibrillary acidic protein
GSK3β: Glycogen synthase kinase 3 beta
HLA-DR: Human leukocyte antigen-DR
IBA1: Ionized calcium-binding adaptor molecule 1
IGFBPL1: Insulin-like growth factor binding protein-like 1
LGN: Lateral geniculate nucleus
MRI: Magnetic resonance imaging
MyD88: Myeloid differentiation primary response 88
NF-κB: Nuclear factor kappa B
NfL: Neurofilament light chain
NLRP3: NLR family pyrin domain containing 3
OCT: Optical coherence tomography
OCTA: Optical coherence tomography angiography
p-tau: Phosphorylated tau
p-tau181: Tau phosphorylated at threonine 181
PLSCR1: Phospholipid scramblase 1
PSD95: Postsynaptic density protein 95
RGC: Retinal ganglion cell
RNFL: Retinal nerve fiber layer
siRNA: Small interfering RNA
TLR4: Toll-like receptor 4
TREM2: Triggering receptor expressed on myeloid cells 2
t-Tau: Total tau
V1: Primary visual cortex
